# Imputation-Based Fine-Mapping Suggests That Most QTL in an Outbred Chicken Advanced Intercross Body Weight Line Are Due to Multiple, Linked Loci

**DOI:** 10.1534/g3.116.036012

**Published:** 2016-10-31

**Authors:** Monika Brandt, Muhammad Ahsan, Christa F. Honaker, Paul B. Siegel, Örjan Carlborg

**Affiliations:** * Department of Medical Biochemistry and Microbiology, Uppsala University, 751 23 Sweden; † Division of Computational Genetics, Department of Clinical Sciences, Swedish University of Agricultural Sciences, 750 07 Uppsala, Sweden; ‡ Department of Animal and Poultry Sciences, Virginia Polytechnic Institute and State University, Blacksburg, Virginia 24061

**Keywords:** imputation-based association, advanced intercross body weight line, Virginia chicken lines, QTL fine-mapping

## Abstract

The Virginia chicken lines have been divergently selected for juvenile body weight for more than 50 generations. Today, the high- and low-weight lines show a >12-fold difference for the selected trait, 56-d body weight. These lines provide unique opportunities to study the genetic architecture of long-term, single-trait selection. Previously, several quantitative trait loci (QTL) contributing to weight differences between the lines were mapped in an F_2_-cross between them, and these were later replicated and fine-mapped in a nine-generation advanced intercross of them. Here, we explore the possibility to further increase the fine-mapping resolution of these QTL via a pedigree-based imputation strategy that aims to better capture the genetic diversity in the divergently selected, but outbred, founder lines. The founders of the intercross were high-density genotyped, and then pedigree-based imputation was used to assign genotypes throughout the pedigree. Imputation increased the marker density 20-fold in the selected QTL, providing 6911 markers for the subsequent analysis. Both single-marker association and multi-marker backward-elimination analyses were used to explore regions associated with 56-d body weight. The approach revealed several statistically and population structure independent associations and increased the mapping resolution. Further, most QTL were also found to contain multiple independent associations to markers that were not fixed in the founder populations, implying a complex underlying architecture due to the combined effects of multiple, linked loci perhaps located on independent haplotypes that still segregate in the selected lines.

Long-term selective breeding of animals and plants for extreme phenotypes has resulted in genetically distinct lines that are a valuable resource for dissecting the genetic architecture of complex traits (Hill 2005). Most traits of interest in animal breeding (*e.g.*, production of eggs or meat, resistance to disease) are influenced by a combination of genetic and environmental factors. Due to their multi-factorial nature and despite the ability to obtain data on both genome-wide genetic markers and phenotypes from large numbers of individuals, it is challenging to disentangle their genetic architecture by analyzing data from the commercial populations. An alternate strategy is to make use of experimental populations resulting from long-term selection experiments, where the focus has been to develop divergent lines from a common base population using more coherent selection criteria. Such populations will display larger phenotypic differences than populations subjected to composite, commercial breeding programs and hence facilitate in-depth studies of the genetic basis underlying the selection response and general genetic architecture of these traits (Andersson and Georges 2004). Given that many of the agriculturally important traits are related to metabolism, feeding-behavior and growth, they also provide a good model for translational studies to decipher the genetic architecture of traits of interest in human medicine, including obesity, eating disorders, and diabetes.

Kemper *et al.* (2012) recently reviewed the literature on the genetic basis of body size and highlighted how complex the genetic architectures of body size are in species with contributions by many loci with large, intermediate, and small individual effects. Also within species, the genetic basis of variations in body size among strains of mice (Valdar *et al.* 2006), breeds of cattle (Saatchi *et al.* 2014), pigs (Yoo *et al.* 2014), and chickens (Van Goor *et al.* 2015) is often polygenic and due to polymorphisms with modest individual effects. Studies of experimental crosses from artificially selected populations with extreme body sizes in the mouse (Bevova *et al.* 2006; Parker *et al.* 2011) and chicken (Sheng *et al.* 2015) using, for example, chromosome substitution strains (Bevova *et al.* 2006) and advanced intercross lines (AILs) (Darvasi and Soller 1995; Besnier *et al.* 2011; Parker *et al.* 2011) have revealed that the responses to selection in these populations has resulted from selection on highly complex and polygenic genetic architectures.

The Virginia lines are experimental populations established in 1957 to study the genetic effects of long-term (>50 generations), divergent, single-trait selection for 56-d high (HWS) or low (LWS) body weight in chickens (Dunnington and Siegel 1996; Márquez *et al.* 2010; Dunnington *et al.* 2013). The lines originated from the same base population, composed by crossing seven partially inbred White Plymouth Rock chicken lines, and today display more than a 12-fold difference in body weight at 56 d of age (Márquez *et al.* 2010; Dunnington *et al.* 2013). In addition to the direct effects of selection on body weight, the selected lines also display correlated selection responses for a range of metabolic and behavioral traits including disrupted appetite, obesity, and antibody response (Dunnington *et al.* 2013).

The Virginia HWS and LWS lines have been used extensively for studying the genetic architecture of body weight and other metabolic traits. These studies have uncovered a number of loci with minor direct effects on body weight, metabolic traits, and body-stature traits by quantitative trait loci (QTL) mapping in an F_2_ intercross (Jacobsson *et al.* 2005; Park *et al.* 2006; Wahlberg *et al.* 2009). Also, a network of epistatic loci has been found to make a significant contribution to long-term selection response through the release of selection-induced additive variation (Carlborg *et al.* 2006; Le Rouzic *et al.* 2007; Le Rouzic and Carlborg 2008). Explorations of the genome-wide footprint of selection by selective-sweep mapping suggests that perhaps >100 loci throughout the genome have contributed to selection response (Johansson *et al.* 2010; Pettersson *et al.* 2013), and many of these contribute to 56 d body weight (Sheng *et al.* 2015).

To replicate and fine-map the body weight QTL inferred in the F_2_ intercross, we developed, genotyped and phenotyped for body weight at 56 d of age (BW56), a nine-generation AIL. This large AIL originated from the same founders as the F_2_ intercross, but was selectively genotyped at a higher resolution (∼1 marker/cM) in nine QTL (Besnier *et al.* 2011). In this population, most of the original minor (Besnier *et al.* 2011) and epistatic (Pettersson *et al.* 2011) QTL were replicated and fine-mapped. These earlier studies analyzed the data using a haplotype-based linkage-mapping approach in a variance-component based model framework to infer single-locus effects (Besnier *et al.* 2011) or a fixed-effect model framework assuming fixed alternative alleles in the two founder lines for detecting epistasis (Pettersson *et al.* 2011). The variance-component model was used in the replication study to avoid the assumption of allelic fixation in the founder lines. By implementing it in a Flexible Intercross Analysis modeling framework (Rönnegård *et al.* 2008), it was expected to improve power when the parental lines carry alleles with correlated effects (*e.g.*, multiple alleles with similar effects).

Although the initial studies mapped QTL under the assumption of fixation, or an effect correlation, of divergent alleles in the parental lines, the results at the same time implied that multiple alleles might be segregating in several of the mapped regions. To this end, the first QTL replication study in the AIL population (Besnier *et al.* 2011) found a large within founder line heterogeneity in the allelic effects. Later the selective-sweep studies, which utilized data from multiple generations of divergently selected and relaxed lines, identified ongoing selection and multiple sweeps in many QTL (Johansson *et al.* 2010; Pettersson *et al.* 2013), as well as extensive allelic purging (Pettersson *et al.* 2013). This allelic heterogeneity challenges attempts to dissect the architecture of the selected trait via, *e.g.*, QTL introgression (Ek *et al.* 2012). Alternative approaches are therefore needed to uncover multi-locus, multi-allelic genetic architectures in QTL and their contributions to the long-term response to directional selection.

In this study, we explore an imputation-based association-mapping strategy for further dissection of previously mapped and replicated QTL (Besnier *et al.* 2011; Pettersson *et al.* 2011). For this, we made use of available high-density (60K SNP-chip) genotypes for founders (Johansson *et al.* 2010; Pettersson *et al.* 2013) and intermediate-density SNP-genotypes in several QTL in the entire nine-generation AIL pedigree. By increasing the marker density in the QTL throughout the AIL by imputation, we aimed to better capture the effects of segregating haplotypes within and between the divergently selected founder populations than with the previously used markers. This aim can be achieved as the original markers genotyped in the AIL were selected to identify high- and low-line derived alleles, and not alleles that segregate within or across the founder lines. By testing for association between imputed markers and body weight, the fine-mapping analyses were less constrained by the original selection of markers and facilitated a more thorough exploration of the genetic architectures of the nine evaluated QTL. A single-marker association analysis was first used to identify regions with candidate associations. These were then simultaneously analyzed using a backward-elimination approach with bootstrapping to identify statistically independent signals that were robust to the effects of markers elsewhere in the genome and the pedigree-structure in the population. In regions where the signals were robust to the pedigree-structure, the results from the single-marker association analysis were used to fine-map the region. Our imputation-based approach replicated most QTL and also improved the resolution in the fine-mapping analyses by not only using the recombination events in the AIL, but also the historical recombinations in the pedigree. We found that several of the original QTL are likely due to the combined effects of multiple linked loci, several of which are segregating in the founder lines of the AIL.

## Materials and Methods

### Animals

The Virginia chicken lines are part of an ongoing selection experiment to study the genetics of long-term, single-trait selection (Márquez *et al.* 2010; Dunnington *et al.* 2013). It was initiated in 1957 from a base population, generated by intercrossing seven partially inbred lines of White Plymouth Rock chickens. From the offspring of the partially inbred lines, resulting from the intercrossing, the birds with the highest and lowest 56 d body weights (with some restrictions), respectively, were selected to produce the high- and low-weight selected lines (HWS and LWS) (Márquez *et al.* 2010; Dunnington *et al.* 2013). Since then, the lines have undergone divergent selection for increased and decreased body weights with one new generation hatched in March of every year.

An AIL was founded by reciprocal crosses of 29 HWS and 30 LWS founder birds from generation 40 (Besnier *et al.* 2011). The mean, sex-averaged 56 d body weights for HWS and LWS at this generation were 1522 g and 181 g, respectively. Repeated intercrossing of birds was used to develop a nine-generation AIL consisting of generations F_0_–F_8_. In each generation, ∼90 birds were bred by paired mating, genotyped, and weighed at 56 d of age (BW56). In total, the AIL population consisted of 1536 F_0_–F_8_ individuals with complete records on pedigree and genotypes (see Genotyping), and 1348 F_2_–F_8_ individuals with juvenile body weight (BW56) records.

### Genotyping

The complete AIL pedigree (1536 birds) had earlier been genotyped in nine selected QTL for 304 SNP-markers that passed quality control as described in Besnier *et al.* (2011). Further, 40 of the founders for the pedigree (20 HWS and 20 LWS) had also earlier been genotyped using a whole genome 60K SNP-chip (Johansson *et al.* 2010; Pettersson *et al.* 2013). The 6607 markers from the SNP-chip that were informative and passed quality control in that study are located in the nine QTL regions targeted in this study. When merging the information from the 60K SNP-chip and the information from the 304 markers genotyped earlier, 55 markers in 40 founders were genotyped using both methods. Out of these 55 markers, 28 markers with genotype inconsistencies between the genotyping technologies were removed during quality control. In total, our analyses were based on 6888 markers, where 40 of the 59 AIL founders had genotypes for all markers, and the remaining individuals in the pedigree had genotypes for 281 markers. Table 1 shows how these markers are distributed across the nine QTL regions.

**Table 1:** Genotyped and imputed markers across the nine analyzed QTL

GGA*^a^*	QTL*^b^*	Start*^c^* (bp)	End*^c^* (bp)	QTL Size (bp)	Markers AIL*^d^*	Markers 60k*^e^*	Markers Total*^f^*	Marker Density*^g^*
1	Growth1	169 634 954	181 087 961	11 453 008	26	504	530	46
2	Growth2	47 929 675	65 460 002	17 530 328	33	667	700	40
2	Growth3	124 333 151	133 581 122	9 247 972	19	395	414	45
3	Growth4	24 029 841	68 029 533	43 999 693	57	1885	1942	44
4	Growth6	1 354 213	13 511 203	12 156 991	23	514	537	44
4	Growth7	85 459 943	88 832 107	3 372 165	14	141	155	46
5	Growth8	33 696 791	39 052 438	5 355 648	5	221	226	42
7	Growth9	10 916 819	35 491 706	24 574 888	76	1397	1473	60
20	Growth12	7 109 709	13 899 993	6 790 285	28	883	911	134

a *Gallus Gallus* Autosome.

b QTL names as in Jacobsson *et al.* (2005).

c Base pair position according to Chicken genome assembly (galGal3) of May 2006.

d Markers as in Besnier *et al.* (2011).

e Markers as in Johansson *et al.* (2010).

f Total markers in *^d^* and *^e^*.

g Markers/Mb.

### Phasing and imputation of markers

All genotyped markers in the QTL (Table 1) were first ordered according to their physical location in the chicken genome assembly of May 2006 (*galGal3*). In the ordered marker set, the SNP-chip markers were evenly distributed in the intervals between the sparser set of markers genotyped across the entire AIL.

Using the software ChromoPhase (Daetwyler *et al.* 2011), we phased and imputed genotypes for the complete set of 6888 markers across the entire AIL pedigree. ChromoPhase first phases large segments of chromosomes, in our case the QTL regions. It then imputes the missing genotypes in the AIL individuals genotyped with the sparse set of markers from the genotype information available in high-density genotyped founders utilizing the pedigree information. It thus predicts both phased haplotypes across the nine studied QTL and genotypes at markers that were only genotyped in a subset of the founder individuals in the pedigree.

### A two-step fine-mapping approach accounting for population structure

Earlier studies have shown that the genetic architecture of body weight is highly polygenic in the Virginia lines (*e.g.*, Siegel 1962a,b; Jacobsson *et al.* 2005; Wahlberg *et al.* 2009; Johansson *et al.* 2010; Besnier *et al.* 2011; Pettersson *et al.* 2011, 2013; Sheng *et al.* 2015). We therefore implemented a forward-selection/backward-elimination procedure with a termination criteria suitable for a polygenic trait in a bootstrap-based framework to correct for population structure in the AIL (Valdar *et al.* 2009; Sheng *et al.* 2015). As all markers with genotypes could not be included in a backward-elimination analysis due to the limited sample size, we first used a forward-selection based single-marker association analysis to identify a smaller set of statistically suggestive independent signals within each QTL region. The backward-elimination analysis (Valdar *et al.* 2009; Sheng *et al.* 2015) was then used to identify associations robust to possible influences of genetic dependencies (linkage or LD) between markers within the QTL or population structure in the AIL (Peirce *et al.* 2008; Cheng *et al.* 2010).

#### Single-marker association analyses:

The *qtscore* function in the GenABEL package (Aulchenko *et al.* 2007) was used to test for association between body weight at 56 d of age and, genotyped or imputed, individual genetic markers within the targeted QTL. The allelic effect of each marker, ${\hat{\beta }}_{\text{genotype}},$ was estimated using a regression model (Model 1), where the genotype at each marker was coded in ***Z*** as 0 if homozygous for the major allele, 1 if heterozygous, and 2 if homozygous for the minor allele. Sex and generation were added as categorical covariates, with two different levels for sex and seven different levels for generation, defined for each individual in ***X***. The phenotype, body weight at 56 d of age, is given in the numerical variable ***y***.

$$y=\mu +\beta_{\text{sex},\text{generation}}X+\beta_{\text{genotype}}Z+\varepsilon \text{(Model 1)}$$

*ε* was assumed to be iid and normally distributed around 0 with variance $\sigma^{2}.$ *μ* is the intercept, which in this model represented the mean body weight at 56 d of age for individual F_2_ females. The associations for the individual markers from this model were used for comparisons to results from earlier linkage-mapping analyses to fine-map the QTL in this pedigree that did not account for the possible effects of pedigree-structure (Model A in Besnier *et al.* 2011). Further, they were also used to evaluate the resolution of regions with associations robust to the pedigree-structure in the population (described in detail in Results).

Next, a forward-selection analysis was performed by scanning across all markers within each QTL using Model 1. If any of the markers were nominally significant (*P* < 0.05) in the scan, the marker with the strongest association was added as a covariate in the model. This procedure was repeated until no additional significant markers were detected. The markers from this analysis with an allele-frequency > 0.10 in the population were subjected to the full backward-elimination analysis described in the next section.

#### A multi-locus association analysis to identify regions with associations that are robust to the pedigree-structure in the population:

In short, we used a bootstrap-based backward-elimination model selection framework (Sheng *et al.* 2015) across the markers selected by forward-selection in the QTL. An adaptive model selection criterion controlling the False Discovery Rate (Abramovich *et al.* 2006; Gavrilov *et al.* 2009) was used during backward-elimination in a standard linear model framework, starting with a full model including the fixed effects of sex and generation, and the additive effects of all markers (Model 2):

$$y=\mu +\beta_{\text{sex},\text{generation},\text{markers}}X+\varepsilon \text{(Model 2)}$$

where phenotype, sex, and generation were coded as described for Model 1 and where *ε* again is assumed to be iid and normally distributed around 0 with variance $\sigma^{2}.$ The intercept, μ, represents the mean body weight at 56 d of age for female individuals from the F_2_ generation. In Model 2, genotypes were coded based on the line-origin of the alleles at each locus. Genotypes of individuals homozygous for the major allele in the AIL founders from the high-weight selection line were coded as 1 at that locus. If an individual was heterozygous, its genotype was coded as 0. Genotypes of individuals homozygous for the allele corresponding to the major allele in AIL founders from the low-weight selection line were coded as −1. By coding genotypes in a −1, 0, and 1 manner, the estimates of the marginal allele-substitution effects, ${\hat{\beta }}_{\text{marker}},$ from fitting Model 2 will be negative if the allele that is at highest frequency in the high-weight line decreases weight or if the allele with highest frequency in the low-weight line increases weight.

Convergence was based on a 20% False Discovery Rate (FDR) level. The analysis was performed using bootstrapping with 1000 resamples. Markers with an RMIP (Resample Model Inclusion Probability) > 0.46, as suggested for an AIL generation F_18_ (Valdar *et al.* 2009), were included in the final model. The FDR in the final model was confirmed using the original FDR procedure described in Benjamini and Hochberg (1995) as implemented in the *p.adjust* function in the R stats-package (R Development Core Team 2015). The additive genetic effect for each locus was estimated using the multi-locus genetic model described above (Model 2). The contribution of a set of *n* associated markers to the founder line difference was calculated as $\sum_{i=1}^{n}\left(2a_{i}\times \left|p_{i}\left(\text{HWS}\right)-p_{i}\left(\text{LWS}\right)\right|\right),$ where $a_{i}$ is the allele-substitution effect for marker *i*, and $p_{i}\left(\text{LWS}\right)$ are the frequencies of the major AIL allele at marker *i* in the HWS and LWS founders, respectively.

### Data availability

Genotype, phenotype, and pedigree data are included in the supplemental files. Supplemental Material, File S1 contains detailed descriptions of all supplemental data files. File S2 contains the genotypes, File S3 the pedigree, and File S4 the phenotypes.

## Results and Discussion

**Figure 1:**
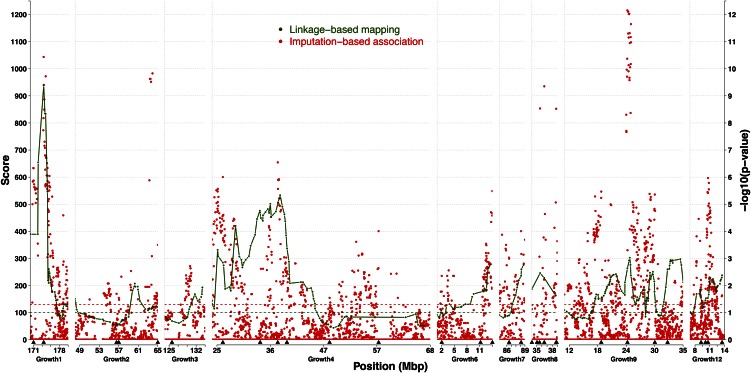
Comparison between linkage- and association-based fine-mapping analyses of nine QTL in an AIL chicken population. Green lines show the statistical support curve (score statistics from Model A) for the linkage-based mapping study of (Besnier *et al.* 2011) and the red dots associations to each analyzed marker in the new imputation-based association analysis (this study). The green and red horizontal dotted lines indicate the experiment-wide significance threshold for earlier linkage-analysis and the nominal significance in the imputation-based association analysis, respectively. Arrowheads under the *x*-axis indicate the position of markers identified as experiment-wide significant (20% FDR) in the bootstrap-based backward-elimination procedure.

We compared the results of the imputation-based association analyses with the previously reported results from the linkage-based analysis of the same nine QTL in Besnier *et al.* (2011). Figure 1 shows the statistical support for association and linkage to BW56 across the QTL. The significances for all the genotyped and imputed markers from the single-marker associations are provided together with the results from model A in Besnier *et al.* (2011) that were also obtained without correction for population structure. Figure 1 also highlights those regions that contain associations robust to the pedigree-structure in the bootstrap-based forward-selection/backward-elimination analyses (Figure 1 and Table 2). Overall, the results from these three analyses overlap well. Together, they show that most regions with strong associations in the single-marker association were robust to the pedigree-structure and that the association analysis approach using imputed genotypes for SNPs suggests that several QTL were likely due to multiple linked loci. In the sections below, these results are described and discussed in more detail.

**Table 2:** Estimated additive effects and standard error for experiment-wide independent association signals, between body weight at 56 d of age and genotype, identified in a bootstrap-based approach implemented in a backward-elimination model selection framework across the markers in the genotyped QTL

GGA*^a^*	QTL*^b^*	Location*^c^* (bp)	Marker*^d^*	ΔAF*^e^*	a ± SE*^f^*	Sign*^g^*
1	Growth1	170 637 618	rs13968052^i^	0.28	16.3 ± 6.6	1.3 × 10^−2^
		173 709 608	rs14916997	0.71	19.3 ± 5.5	4.8 × 10^−4^
2	Growth2	56 720 515	rs14185295	0.32	13.2 ± 6.2	3.3 × 10^−2^
		57 198 629	rs14185836^i^	0.16	−12.1 ± 6.2	5.4 × 10^−2^
		65 460 002	rs14196021	0.32	14.0 ± 5.9	1.7 × 10^−2^
2	Growth3	126 000 254	rs16120360	0.36	12.6 ± 5.8	3.0 × 10^−2^
3	Growth4	26 215 175	rs14328509	0.53	19.8 ± 5.4	2.3 × 10^−4^
		33 743 569	rs314044798^i^	0.46	−21.9 ± 6.1	3.8 × 10^−4^
		37 287 334	rs316425755^i^	0.12	27.8 ± 6.9	5.7 × 10^−5^
		39 139 081	rs15468467^i^	0.23	27.0 ± 7.3	2.1 × 10^−4^
		47 729 342	rs316384373^i^	0.30	−13.1 ± 6.8	5.5 × 10^−2^
		57 624 596	rs14363139^i^	0.64	18.2 ± 4.1	2.9 × 10^−4^
4	Growth6	2 392 397	rs14419462^i^	0.30	19.7 ± 7.6	1.0 × 10^−2^
		10 914 312	rs14428120^i^	0.77	−14.5 ± 5.8	1.3 × 10^−2^
		13 511 203	rs15500313	0.78	17.7 ± 6.0	3.4 × 10^−3^
4	Growth7	86 755 267	rs14499758	0.32	18.1 ± 6.3	3.9 × 10^−3^
		88 325 118	rs15639000	0.06	13.4 ± 5.4	1.3 × 10^−2^
5	Growth8	33 713 055	rs14530756^i^	0.46	−13.5 ± 5.3	1.1 × 10^−2^
		34 772 650	rs16487762^i^	0.53	15.0 ± 6.8	2.7 × 10^−2^
		35 299 978	rs16487933^i^	0.40	−18.6 ± 6.0	1.8 × 10^−3^
		36 291 277	rs13585490	0.15	14.1 ± 5.3	7.3 × 10^−3^
		38 774 986	rs315605733^i^	0.23	23.3 ± 7.3	1.4 × 10^−3^
		38 867 279	rs314075508^i^	0.37	16.4 ± 5.5	3.0 × 10^−3^
7	Growth9	18 544 622	rs14611566^i^	0.00	−20.1 ± 5.1	9.4 × 10^−5^
		23 959 214	rs16596357^i^	0.17	18.9 ± 5.6	7.7 × 10^−4^
		29 631 963	rs10727581^i^	0.32	15.9 ± 6.1	9.0 × 10^−3^
		32 262 733	rs317586448^i^	0.33	−13.2 ± 5.0	8.7 × 10^−3^
20	Growth12	9 302 754	rs14277526^i^	0.34	21.8 ± 6.8	1.4 × 10^−3^
		10 165 171	rs14278292^i^	0.37	−14.7 ± 6.5	2.4 × 10^−2^
		10 667 729	rs16172598^i^	0.56	14.3 ± 5.1	5.1 × 10^−3^
		13 427 530	rs16176151^i^	0.13	8.5 ± 5.2	1.0 × 10^−1^

For a marker with a positive estimated additive effect, the effect on weight is caused by the allele with its origin in the line associated with the sign of the effect, *i.e.*, an allele with its origin in high-line is associated with an increase in body weight and an allele with its origin in low-line is associated with a decrease in body weight. In cases where a weight-increasing allele has its origin in the low-line or a weight-decreasing allele has its origin in the high-line the sign of the estimated additive effect will be negative.

a *Gallus Gallus* Autosome.

b QTL name as in Jacobsson *et al.* (2005).

c Base pair position according to Chicken genome assembly (galGal3) of May 2006.

d SNP name as in NCBI dbSNP where imputed markers are labeled with ^i^ after the marker name.

e Difference in allele-frequency between the HWS and LWS founder lines.

f Additive effect ± SE calculated in a model including all loci in the table.

g Significance of the estimated additive genetic effect in a model including all loci in the table.

### Four statistically independent associated markers in the GGA7 QTL Growth9

The QTL *Growth9* on GGA7 (*Gallus Gallus* Autosome 7) (Figure 1; 10.9–35.5 Mb) was the only QTL that reached genome-wide significance in the first F_2_ intercross between the HWS and LWS lines (Jacobsson *et al.* 2005). It was later identified as a central QTL in an epistatic network explaining a large part of the difference in weight between HWS and LWS lines (Carlborg *et al.* 2006). In the earlier fine-mapping analysis, the linkage signal covered most of the QTL region (from 15 to 35 Mb), but subsequent analyses showed that two independent loci were segregating in the region (Besnier *et al.* 2011). The signal in the imputation-based association analysis performed here is more focused, with a highly significant signal in a 2.8 Mb region between 23.7 and 26.4 Mb. This region overlaps with the strongest signal in the linkage-scan and is tagged by a single imputed marker (*rs16596357^i^*; Table 2) in the multi-locus analysis accounting for population structure. The major allele in the HWS line (*P* = 0.67) increases weight by 18.9 (SE 5.6) g, and it still segregates at an intermediary frequency (*P* = 0.50) in the LWS line. Previously, Ahsan *et al.* (2013) explored potential candidate mutations in the QTL and found two regulatory SNPs near the peak at 21 Mb (21.6 and 22.7 Mb) and a synonymous-coding SNP in a CpG island in an exon of the Insulin-like growth factor binding protein 2 (*IGFBP2*) gene in the middle of the major association peak at 24.8 Mb. In addition to the strong association around 24 Mb, the association analysis also highlights two additional regions (centered around 18 and 29 Mb). A single imputed marker (*rs14611566^i^*; Table 2) is retained in the first region in the multi-locus analysis accounting for population structure. This marker has an estimated allele-substitution effect of −20.1 (SE 5.1) g, but as it segregates at equal, intermediary frequencies in the HWS and LWS lineages (*P* = 0.50) it did not contribute to the founder line difference. Two linked imputed markers are kept in the third region (*rs10727581^i^*; *rs317586448^i^*; 2.6 Mb apart; Table 2). Here, the first marker is nearly fixed for one allele in the LWS line (*P* = 0.93), but segregates at an intermediary frequency in the HWS line (*P* = 0.61). At the second marker, the major allele in the HWS (*P* = 0.74) segregates at an intermediary frequency in the LWS (*P* = 0.41). Due to the close linkage between the associated markers, it is difficult to interpret their individual effects and disentangle whether the detected associations are due to the LD-pattern of multiple closely linked loci or a single locus with multiple segregating alleles. The peak in the F_2_ QTL overlaps the major 23.7–26.4 Mb association peak detected in this analysis (Wahlberg *et al.* 2009). Due to the low differences in allele frequencies between the associated markers in the three regions, their total contribution to the founder line difference is small (8 g) amounting to only about 10% of the original estimated F_2_ QTL effect of 86 g (Wahlberg *et al.* 2009).

### Two statistically independent associated markers in the GGA1 QTL Growth1

The strongest association in the study by Besnier *et al.* (2011) was found on GGA1 in the QTL *Growth1* (Figure 1; 169.6–181.1 Mb). Here, the second strongest association was detected in that QTL. The imputation-based association analysis highlights two significant associations separated by a region of very low association and both associations remained in the multi-locus analysis accounting for population structure (the imputed marker *rs13968052^i^* and the genotyped marker *rs14916997* at 170.6 and 173.7 Mb, respectively). The strongest of these association-peaks was located near the peak detected using the earlier linkage-based analysis. Several of the significant associated markers were located in this region (173.6–175.3 Mb). A candidate gene for growth, Asparagine-linked glycosylation 11 homolog gene (*ALG11*), is located at 174.6 Mb and has a strong mutation in its regulatory region (Ahsan *et al.* 2013). The second association was found to a group of significant markers in a narrower region upstream from the main linkage-peak (170.3–171.7 Mb). The association analysis thus suggests that the original 10.6 Mb QTL region that has its peak between markers located at 175.2–177.7 Mb is due to the effects of two separate loci located in these confined 1.5 and 1.8 Mb regions. As the two associated markers are closely linked in this population, it is difficult to interpret their individual effects, but their total contribution to the founder line difference (37 g) is about 75% of that by the original *Growth1* QTL as estimated in the F_2_ analysis (49 g; Wahlberg *et al.* 2009).

### Four statistically independent associated markers in the GGA2 QTL Growth2 and Growth3

GGA2 contains two QTL, *Growth2* (Figure 1; 47.9–65.5 Mb) and *Growth3* (Figure 1; 124.3–133.6 Mb). The multi-locus analyses identified three significantly associated markers in *Growth2*, where the first two are clustered at 56.7 and 57.2 Mb (genotyped marker *rs14185295* and imputed marker *rs14185836^i^*, respectively), with the last genotyped marker (*rs14196021*) located at 65.5 Mb. The distance between the markers, and the region of low association between them in the single-marker analysis (Figure 1), suggests that two linked loci contribute to the *Growth2* QTL. In the earlier linkage-based analysis the strongest signal in *Growth2* was located in between these markers at 60.6 Mb. The QTL-peak in the original F_2_ analysis (Jacobsson *et al.* 2005) is difficult to assess as nearest marker (*MCW130*) is not mapped to the chicken genome and no significant signal was found using a denser marker-map by Wahlberg *et al.* (2009). As the first two markers in the QTL are tightly linked, it is difficult to interpret the individual estimates of their effects; however, the third marker located 8 Mb upstream from them has a small independent effect. The estimated contribution by these loci to the founder line difference is small (14 g), which amounts to about 30% of the original contribution by the *Growth2* QTL in the F_2_ analyses (Jacobsson *et al.* 2005). In *Growth3*, a single association was detected to a genotyped marker (*rs16120360*) in the multi-locus analysis and this peak was inside the original F_2_ QTL [101.6–131.9 Mb; (Jacobsson *et al.* 2005)] but shifted almost 4 Mb upstream from the top signal found in the earlier linkage-based analysis. The contribution by this marker to the line difference is small (9 g) and about 15% of that in the original F_2_ analysis (Jacobsson *et al.* 2005).

### Six statistically independent associated markers in the GGA5 QTL Growth8

One of the strongest association signals was found on GGA5 in the QTL *Growth8* (Figure 1; 33.7–39.1 Mb) and six markers were retained in this region after accounting for population structure in the multi-locus analysis (Table 2). The single-locus analysis suggests that these markers tag two loci – one from 33.7–36.3 Mb (three imputed and one genotyped marker; Table 2) and one around 38.8 Mb (two imputed markers). The markers were located between the markers flanking the original QTL (21.6–44.2 Mb) in Jacobsson *et al.* (2005) and overlaps with the earlier linkage signal. The association signal was, however, stronger than the linkage signal suggesting that the imputed markers tag the QTL better than the haplotypes inferred from the sparser set of genotyped markers. Although the linkage between the markers again makes it difficult to obtain stable estimates for the effects of individual markers in the two associated loci, their estimated contribution to the founder line difference (16 g) amounts to about one third of that estimated effect in the F_2_ (Jacobsson *et al.* 2005).

### Six statistically independent associated markers in the GGA3 QTL Growth4

In the QTL *Growth4* on GGA3 (Figure 1; 24.0–68.1 Mb), both the association and linkage analyses identify a broad region of association from 24 to 41 Mb. Although the statistical support curve in the linkage analysis contains multiple peaks, that analysis was unable to fine-map the region into multiple, independent signals. Here, the multi-locus analysis suggests that perhaps up to four independent regions contribute to this QTL, with one associated genotyped marker at 26.2 Mb, three imputed markers from 33.7–39.1 Mb, one imputed marker at 47.7 Mb, and one at 57.6 Mb. The single-locus association analysis highlights two particularly strong and distinct association-peaks located approximately between 24–27 and 33–37 Mb, respectively. A candidate mutation in *Growth4* was found near the second association region at 33.6 Mb inside the regulatory region of Cysteine rich transmembrane BMP regulator 1 (*CRIM1*) (Ahsan *et al.* 2013). The associated region around 55–57 Mb displayed very low significance in the previous linkage analysis. The outmost markers (26.2 and 57.6 Mb) have allele-substitution effects of 19.8 (SE 5.4) and 18.2 (SE 4.1) g, respectively, and are rather diverged between the lines (∼50% difference between the lines). For the other two clusters of markers, it is difficult to obtain stable estimates of their individual effects. Their estimated joint contribution to the founder line difference (35 g) is about 65% of that in the original F_2_ analysis (Jacobsson *et al.* 2005).

### Four statistically independent associated markers in the GGA20 QTL Growth12

The earlier linkage analysis replicated the QTL *Growth12* on GGA20 (Figure 1; 7.1–13.9 Mb), with the strongest associated marker at 10.7 Mb, and the signal covered most of the region (8–13.9 Mb). Four markers were significant in the multi-locus analysis and three imputed markers were located in the main single-marker association peak covering the region from 9 to 11 Mb, while the fourth associated imputed marker was located about 4 Mb upstream (13.4 Mb). Again, it is difficult to interpret the individual effects of the tightly linked markers; however, their estimated contribution to the line difference (22 g) is about 75% of that estimated in the F_2_ analysis (Jacobsson *et al.* 2005).

### Five statistically independent associated markers in the GGA4 QTL Growth6 and Growth7

In both *Growth6* (Figure 1; 1.3–13.6 Mb) and *Growth7* (Figure 1; 85.4–88.9 Mb) on GGA4, several markers were significant in the multi-marker analysis. The single-marker analysis illustrates that these markers tag association-peaks that were located very close to the main peaks in the earlier linkage-based analysis, suggesting that both analyses identified the same underlying loci. The association analysis identified two genotyped markers in a region in *Growth7* with strong association around 86.5–88.5 Mb. It is difficult to interpret their individual effects due to the close linkage, but their estimated contribution to the founder line difference was small (13 g) amounting to about a fifth of that estimated in the F_2_ population (66 g; Jacobsson *et al.* 2005). In *Growth6*, the multi-marker analysis detected associations to two imputed and one genotyped marker representing one locus at 2.3 Mb and a second locus at 10.9–13.5 Mb. Here, the tight linkage in the second locus also makes interpretation of their individual effects difficult, whereas the allele frequencies are not that differentiated in the first locus. Their estimated contribution to the line difference is therefore small (17 g) amounting to about a fifth of the 92 g estimated in the original F_2_ analysis (Jacobsson *et al.* 2005).

### General comments

Here we report the results from using an imputation-based association-mapping strategy to fine-map QTL in a nine-generation, outbred AIL. By combining high-density genotyping of the AIL founders with imputation throughout the rest of the pedigree utilizing a sparser genotyped marker backbone, we increased the marker density ∼20-fold in the studied regions. This subsequent association analysis had a comparable power for replication of QTL to the earlier used linkage-based strategy. In addition to this, the new analyses also detected multiple association-peaks in several of the QTL and narrowed the associated regions considerably compared to the regions detected previously (Besnier *et al.* 2011). Together, they suggest that this imputation-based association-mapping approach is a promising strategy for improving the resolution in fine-mapping studies in outbred pedigrees, where high-density marker genotypes are not available for all studied individuals. When interpreting the full results from the multi-locus backward-elimination analysis (Table 2), it should be noted that the results are reported at a 20% False Discovery Rate. Although a significant proportion of the markers could thus be false positive associations, as illustrated by the individual P-values for the additive effects of the associated markers, most of the peaks on the chromosomes also contain markers with more significant individual associations. We used this threshold because earlier mapping and replication studies confirmed that the QTL contain at least one small-effect locus contributing to 56 d weight, and that the major aim of this study was to provide an overall view of the most likely genetic architecture of the fine-mapped QTL, rather than high-confidence estimates for the individual regions.

In both *Growth1* and *Growth4* two strong, distinct association signals were identified. Also in the QTL *Growth8* and *Growth9* the new analysis identified strong association-peaks covering many markers. In these regions, the strongest linkage signals identified in the previous fine-mapping analysis (Besnier *et al.* 2011) overlap with the strongest signals in the current analyses. However, the association analysis also separates the signals into multiple peaks and highlights narrower regions. Hence, it provides more useful input for further analyses to identify candidate genes underlying the QTL. In most cases the associated regions are restricted to distinct 2–3 Mb regions, which as indicated by the findings from Ahsan *et al.* (2013), is useful for restricting the bioinformatics analyses to only the most promising candidate genes for further functional studies. In *Growth6*, *Growth7*, and *Growth12*, the association signals were not as significant as in the other QTL. Despite this, the multi-locus analyses suggest that the linkage signals in the earlier analyses were due to distinct loci with independent effects, mapped here into narrower association-peaks.

Overall, the location of the association signals in this study overlapped well with the top signals in the earlier linkage analyses. However, in two of the QTL (*Growth2* and *Growth3*), the association-peaks are shifted when comparing results from both studies. Further work is needed to explore whether this reflects separate loci with distinct genetic architectures that could only be detected with the respective methods, or if they reflect a signal of the same underlying causal locus.

Here, we estimated the additive genetic effects of the fine-mapped regions using data from the F_2_–F_8_ generations of the AIL. To evaluate whether they were in general agreement with estimates obtained for the same regions in earlier studies, we compared them to the estimates obtained from our first large F_2_ population (Jacobsson *et al.* 2005; Wahlberg *et al.* 2009). The QTL effects were generally lower in the F_2_–F_8_ data than in the F_2_. Although this may be interpreted as the F_2_ estimates being inflated, several other factors could also be considered. First, the 56 d body weights were considerably lower for the F_8_ generation because younger dams were used to generate these (Besnier *et al.* 2011). In the analyses, a fixed effect of generation was used to account for the mean weight differences between generations. However, it did not account for the likely scenario that the QTL effects were smaller in these F_8_ birds due to their lower body weight. As about 30% of the birds in the pedigree are from this generation, this would bias the overall effects downward. Standardization of the phenotypes from different generations to the same mean and variance is a way to possibly account for this, but a caveat of that approach is an introduction of an upward bias of the effects if the QTL effects in the F_8_ are, in fact, not that much smaller. We therefore chose to report the more conservative estimates based on analyzing the nonstandardized phenotypes. Second, eight of the nine QTL contain fine-mapped regions with associations to several tightly linked markers. If these markers are located on the same haplotypes, it is not possible to disentangle their effects in this pedigree as too few recombination events have accumulated in the F_2_–F_8_ generations of the AIL, and due to such collinearities the estimates for the individual markers reported here would not properly describe the contributions of these haplotypes to the line difference. In several of the regions with multiple associated markers, the estimates of the additive effects were also negative for at least one of these markers. Although this could be interpreted as transgression being common in the population, we find them more likely to result from the collinearities among the closely linked markers. Further analyses utilizing, for example, later AIL generations, markers that specifically tag the haplotype-structure of the founder lines and methods that can account for multi-allelic genetic architectures will be needed to disentangle the genetic architectures of these loci and quantify their contributions to the founder line difference. Third, the F_2_ QTL estimates were obtained using a line-cross analysis where it is assumed that the founder lines are fixed for alternative QTL alleles (Jacobsson *et al.* 2005; Wahlberg *et al.* 2009). In the current association analysis, it is instead assumed that the alternative alleles at the tested markers tag nearby functional alleles. As none of the associated markers were fixed for alternative alleles in the founder lines (Table 2 and Table S1), the current fine-mapping analysis suggests that one, or both, founder lines segregate for multiple functional alleles in the QTL. To compare the estimates from the line-cross analysis in the F_2_ and the association analyses in the AIL F_2_–F_8_ generations, they need to be compared using a common reference. Here, we did this by estimating how much the associated markers in each QTL were expected to contribute to the founder line difference under the assumption that they act completely additively. Under this assumption, their contribution would equal twice the sum of the allele-substitution effects of the markers in a QTL weighted by their respective allele-frequency differences between the founder lines. That is, if the markers are fixed for alternative alleles in the founder lines they would contribute two allele-substitution effects to the founder line difference, whereas they would contribute nothing if the allele was present at equal frequencies in both founder lines. This estimate is conservative as, for example, dominance leads to an underestimation of the contribution of the locus. This as, in the presence of a dominant allele, one line will not need to be fixed to contribute most of its effect because this is also displayed in the heterozygotes for that allele. When comparing estimates this way, the combined effects of the associated markers in each of the QTL contribute from 10 to 75% of that estimated in the F_2_ by Jacobsson *et al.* (2005). In total, the QTL replicated here contributed 171 g to the founder line difference, compared to the 416 g in the F_2_. As discussed above, further analyses in other populations with more informative genetic markers using other statistical methods are, however, required to explore this further.

Our analyses suggest that there is extensive within-line segregation in the QTL regions. One possible explanation for the slow fixation in these loci could be that the beneficial alleles at the linked fine-mapped loci were located on different haplotypes at the onset of selection. Due to the low selection pressure on each QTL region resulting from the highly polygenic architecture of the selected trait, the close linkage between the loci contributing to the QTL, and the small effective breeding population, the probability that beneficial recombinant haplotypes are selected and increase in frequency in the population should be low. Another alternative explanation could be that the effects of the linked loci are dependent on the genetic background (epistasis) or dominance, which might have affected the selection pressure on individual contributing loci. As we did not explore the contributions by dominance or epistasis in this study, further work would be necessary to evaluate their contributions to the low fixation in the QTL.

A key for successful imputation of the high-density marker set throughout the AIL pedigree is that the haplotypes across these markers are correctly estimated in the founders. There are several properties of the Virginia lines that improve haplotype estimation from high-density genotypes. First, as the number of generations since the lines diverged is relatively few (40 generations), most new haplotypes will result from recombination of original haplotypes, rather than by new mutations. Second, the strong artificial selection imposed on the populations since they were founded is likely to have further reduced haplotype diversity across the genome. This is likely the reason that many selective-sweeps across long haplotypes have been found to be fixed, or nearly fixed, across the genome within and between the lineages (Johansson *et al.* 2010; Pettersson *et al.* 2013). This is reflected in a large average LD-block size (> 50 kb) across the genome (Marklund and Carlborg 2010). Given the density of the 60k SNP-chip genotyping used here, several markers will be present on each such LD-block and hence improve efficiency in haplotype estimation. Additional genotyping will, however, be necessary in subsequent generations to experimentally confirm the associations to imputed markers reported here.

Genotype data are available for all individuals in the AIL pedigree. The dense marker backbone (∼1 marker/cM) from the first genotyping of the AIL (Besnier *et al.* 2011), allow the relatively long haplotypes that are inherited as intact segments from parents to offspring to be efficiently phased, imputed, and traced throughout the pedigree for later association analyses.

The highly polygenic genetic architecture in this population is consistent with what has been revealed in other fine-mapping analyses in deep intercrosses (Parker *et al.* 2011) and chromosomal substitution strains (Bevova *et al.* 2006) involving intensively selected mouse populations. Recent work on a mouse population that has evolved to an extreme body size in nature has also uncovered a highly polygenic architecture of adaptation (Gray *et al.* 2015), illustrating that complex genetic architectures are likely to be involved in responses to both natural and experimental selection. Further, our detection of multiple associations to nearby markers in our AIL is also consistent with reports from other AIL-based fine-mapping studies in chickens from outbred base-populations (Van Goor *et al.* 2015) and association studies within and across cattle breeds (Saatchi *et al.* 2014). Subsequent studies will help to elucidate whether the underlying genetic architecture of associations detected to linked markers in this and other outbred populations are primarily due to the segregation of multiple haplotypes in the outbred founder populations and breeds or a reflection of several tightly linked functional polymorphisms.

Here, the association analysis was performed using a linear model including fixed effects of genotype, sex, and AIL generation. Sex and generation were included as both these environmental factors had significant effects on BW56 (Besnier *et al.* 2011). Implementing the model selection by backward-elimination in a bootstrap-based framework is a way to account for possible effects of population structure in the AIL that might increase the risk for reporting false positives. However, since the association signals in most cases overlap well with the final marker set resulting from the testing of experiment-wide significant associations, we do not find this to be any cause of great concern in this experiment.

### Conclusions

In conclusion, this study shows that the proposed imputation-based association-mapping strategy, and further model selection by backward-elimination in a bootstrap-based framework, is useful for identifying independent association signals within and across the nine evaluated QTL. The association-peaks were narrower than those obtained in the earlier performed linkage analysis, often highlighting regions down to 2–3 Mb in length allowing the identification of multiple association signals in several QTL. This suggests that the association-based strategy has higher resolution, as well as provides an improved power to disentangle the effects of multiple linked loci inside QTL, compared to linkage-based fine-mapping. Combining traditional linkage-based approaches to analyze outbred advanced intercross populations with imputation-based association-mapping approaches might thus be an important and cost-effective approach to improve the efficiency in postassociation bioinformatics analyses and functional explorations aiming to identify candidate mutations. A previous candidate gene study based on the nine QTL fine-mapped here has already reported some interesting mutations in growth-related genes (Ahsan *et al.* 2013) overlapping with the association signals reported here. Further bioinformatics investigations of the regions fine-mapped here could potentially reveal new important genes and mutations affecting body weight in these chicken lines and provide new candidate genes for studying the genetic architecture of metabolic traits in other species, including humans.

## Supplementary Material

Supplemental material is available online at www.g3journal.org/lookup/suppl/doi:10.1534/g3.116.036012/-/DC1.
