# Supplementary material for: Imputation-Based Fine-Mapping Suggests That Most QTL in an Outbred Chicken Advanced Intercross Body Weight Line Are Due to Multiple, Linked Loci
Source: G3 (Bethesda). 2016 Oct 31;7(1):119–28. doi: 10.1534/g3.116.036012 (PMC5217102; doi:10.1534/g3.116.036012)
Supplement: Supplementary file 1 [file 119FileS1.docx]

**File S1** This document contains the full legends for the supplemental files. (.pdf, 21 KB)

Available for download as a .pdf file at [www.g3journal.org/lookup/suppl/doi:10.1534/g3.116.036012/-/DC1/FileS1.pdf](http://www.g3journal.org/lookup/suppl/doi:10.1534/g3.116.036012/-/DC1/FileS1.pdf)
