# Supplementary material for: Imputation-Based Fine-Mapping Suggests That Most QTL in an Outbred Chicken Advanced Intercross Body Weight Line Are Due to Multiple, Linked Loci
Source: G3 (Bethesda). 2016 Oct 31;7(1):119–28. doi: 10.1534/g3.116.036012 (PMC5217102; doi:10.1534/g3.116.036012)
Supplement: Supplementary file 2 [file 119FileS1.pdf]

FileS1.txt

Tab delimited text-file with genotypes for 1536 individuals and 6888 markers included in the study. The first column represents the animal id with the generation included. The following columns gives the genotypes for all individuals at each genotyped marker, the column name corresponds to the current marker. Missing genotypes are coded as ZZ.

FileS2.txt

Tab delimited text-file with the complete pedigree for all 1536 individuals included in the study. Columns are ordered in the following way, individual id, father id for current individual, mother id for current individual.

FileS3.txt

Tab delimited text-file with individual ID, sex, generation and bodyweight at 56 days of age listed for all 1348 individuals included in the association analysis. Females are coded as 0 and males as 1.
