# Supplementary material for: Imputation-Based Fine-Mapping Suggests That Most QTL in an Outbred Chicken Advanced Intercross Body Weight Line Are Due to Multiple, Linked Loci
Source: G3 (Bethesda). 2016 Oct 31;7(1):119–28. doi: 10.1534/g3.116.036012 (PMC5217102; doi:10.1534/g3.116.036012)
Supplement: Supplementary file 5 [file 119FileS3.docx]

**File S3** Tab delimited text-file with the complete pedigree for all 1536 individuals included in the study. (.zip, 36 KB)

Available for download as a .zip file at [www.g3journal.org/lookup/suppl/doi:10.1534/g3.116.036012/-/DC1/FileS3.zip](http://www.g3journal.org/lookup/suppl/doi:10.1534/g3.116.036012/-/DC1/FileS3.zip)
