# Supplementary material for: Imputation-Based Fine-Mapping Suggests That Most QTL in an Outbred Chicken Advanced Intercross Body Weight Line Are Due to Multiple, Linked Loci
Source: G3 (Bethesda). 2016 Oct 31;7(1):119–28. doi: 10.1534/g3.116.036012 (PMC5217102; doi:10.1534/g3.116.036012)
Supplement: Supplementary file 7 [file 119FileS4.docx]

**File S4** Tab delimited text-file with individual ID, sex, generation and bodyweight at 56 days of age listed for all 1348 individuals included in the association analysis. (.zip, 23 KB)

Available for download as a .zip file at [www.g3journal.org/lookup/suppl/doi:10.1534/g3.116.036012/-/DC1/FileS4.zip](http://www.g3journal.org/lookup/suppl/doi:10.1534/g3.116.036012/-/DC1/FileS4.zip)
