# Supplementary material for: Imputation-Based Fine-Mapping Suggests That Most QTL in an Outbred Chicken Advanced Intercross Body Weight Line Are Due to Multiple, Linked Loci
Source: G3 (Bethesda). 2016 Oct 31;7(1):119–28. doi: 10.1534/g3.116.036012 (PMC5217102; doi:10.1534/g3.116.036012)
Supplement: Supplementary file 9 [file 119TableS1.docx]

**Table S1** Statistically suggestive independent signals within each QTL identified as nominally significant (p < 0.05) in the forward-selection procedure. (.pdf, 97 KB)

Available for download as a .pdf file at [www.g3journal.org/lookup/suppl/doi:10.1534/g3.116.036012/-/DC1/TableS1.pdf](http://www.g3journal.org/lookup/suppl/doi:10.1534/g3.116.036012/-/DC1/TableS1.pdf)
