# Supplementary material for: Imputation-Based Fine-Mapping Suggests That Most QTL in an Outbred Chicken Advanced Intercross Body Weight Line Are Due to Multiple, Linked Loci
Source: G3 (Bethesda). 2016 Oct 31;7(1):119–28. doi: 10.1534/g3.116.036012 (PMC5217102; doi:10.1534/g3.116.036012)
Supplement: Supplementary file 10 [file 119TableS1.pdf]

Table S1. Statistically suggestive independent signals within each QTL identified as nominally significant ($p < 0.05$) in the forward-selection procedure. For each QTL, Model 1 was first fitted for all markers and the most significant marker was selected and added as a covariate before refitting model 1 again. This procedure, selecting the most significant marker as a covariate and refitting Model 1, was repeated until no markers within the QTL reached the nominal significance threshold. QTL that were selected at 20% FDR in the following backward-elimination procedure (Table 2 in main manuscript) are highlighted in bold italics.

GGA[a]	QTL[b]	Position[c] (bp)	Marker[d]	a ± SE[e]	Sign[f]	p_{HWS}[g]	p_{LWS}[h]	MAF F_2-F_8[i]
1	Growth1	***173 709 608***	***rs14916997***	***-36.6 ± 5.5***	***3.6×10^{-11}***	***0.11***	***0.82***	***0.48***
		179 613 655	rs313927704	41.6 ± 12.2	6.5×10^{-4}	0.30	0.04	0.05
		170 637 618	***rs13968052***	***19.1 ± 6.6***	***3.8×10^{-3}***	***0.35***	***0.07***	***0.21***
		176 383 356	rs13975251	19.7 ± 7.7	1.1×10^{-2}	0.43	0.09	0.12
		178 029 363	rs317567034	-31.6 ± 11.6	6.4×10^{-3}	0.20	0.02	0.05
		179 806 129	rs314200699	28.8 ± 12.2	1.8×10^{-2}	0.04	0.14	0.05
		169 810 898	rs15487296	161.7 ± 78.5	4.0×10^{-2}	0.00	0.34	0.00
2	Growth2	64 317 336	rs14194723	-101.5 ± 15.9	1.5×10^{-10}	0.04	0.04	0.03
		62 837 662	rs14192893	458.6 ± 138.1	9.0×10^{-4}	0.02	0.00	0.00
		59 778 572	rs316345242	-28.7 ± 9.2	1.8×10^{-3}	0.20	0.02	0.09
		56 720 515	***rs14185295***	***15.3 ± 5.5***	***5.2×10^{-3}***	***0.46***	***0.14***	***0.39***
		54 865 875	rs14183473	57.4 ± 20.3	4.7×10^{-3}	0.00	0.30	0.02
		57 198 629	***rs14185836***	***-14.1 ± 5.4***	***9.7×10^{-3}***	***0.41***	***0.25***	***0.39***
		65 460 002	***rs14196021***	***14.4 ± 6.0***	***1.7×10^{-2}***	***0.43***	***0.11***	***0.25***
		55 330 630	rs15100427	13.7 ± 6.4	3.2×10^{-2}	0.37	0.34	0.21
		62 639 245	rs15107150	-74.8 ± 36.2	3.9×10^{-2}	0.11	0.00	0.00
2	Growth3	130 133 780	rs14248248	20.2 ± 6.5	1.9×10^{-3}	0.07	0.57	0.21
		130 592 117	rs16133110	-62.2 ± 25.4	1.4×10^{-2}	0.00	0.11	0.01
		126 000 254	***rs16120360***	***13.8 ± 6.1***	***2.3×10^{-2}***	***0.54***	***0.18***	***0.28***
3	Growth4	***37 287 334***	***rs316425755***	***-32.6 ± 6.4***	***2.9×10^{-7}***	***0.26***	***0.38***	***0.23***
		26 215 175	***rs14328509***	***-21.8 ± 5.4***	***5.1×10^{-5}***	***0.26***	***0.79***	***0.45***
		28 981 759	rs313368484	92.5 ± 24.7	1.8×10^{-4}	0.02	0.16	0.01
		36 103 180	rs13723817	26.3 ± 6.8	1.0×10^{-4}	0.02	0.55	0.18
		57 624 596	***rs14363139***	***-18.0 ± 5.2***	***5.7×10^{-4}***	***0.13***	***0.77***	***0.50***
		61 227 711	rs16284878	-34.6 ± 11.0	1.6×10^{-3}	0.59	0.09	0.06
		47 729 342	***rs316384373***	***21.9 ± 6.9***	***1.6×10^{-3}***	***0.04***	***0.34***	***0.16***
		53 589 855	rs14359071	34.7 ± 11.9	3.5×10^{-3}	0.37	0.27	0.05
		60 099 912	rs14364587	-33.2 ± 11.2	3.0×10^{-3}	0.57	0.00	0.06
		39 139 081	***rs15468467***	***-16.6 ± 6.4***	***9.2×10^{-3}***	***0.15***	***0.38***	***0.21***
		33 743 569	***rs314044798***	***15.6 ± 5.0***	***1.9×10^{-3}***	***0.09***	***0.55***	***0.38***
		40 789 962	rs16256006	-53.8 ± 20.0	7.2×10^{-3}	0.04	0.00	0.01
		38 049 506	rs14341458	72.7 ± 26.2	5.6×10^{-3}	0.09	0.00	0.01
		36 036 392	rs14339632	29.3 ± 10.7	6.1×10^{-3}	0.00	0.11	0.05
		53 236 895	rs13570951	-17.9 ± 7.1	1.2×10^{-2}	0.52	0.05	0.10
		49 677 389	rs15350215	55.0 ± 21.0	8.9×10^{-3}	0.17	0.00	0.01
		24 558 483	rs14326932	-180.3 ± 75.5	1.7×10^{-2}	0.00	0.20	0.00
		52 685 153	rs13570803	14.0 ± 5.8	1.7×10^{-2}	0.33	0.68	0.20
		61 496 004	rs315203852	24.7 ± 10.3	1.6×10^{-2}	0.57	0.00	0.06
		25 074 560	rs14327473	41.5 ± 19.5	3.3×10^{-2}	0.22	0.11	0.02
		50 259 236	rs14356142	-46.7 ± 22.5	3.8×10^{-2}	0.00	0.16	0.01
4	Growth6	***13 511 203***	***rs15500313***	***25.4 ± 5.5***	***3.3×10^{-6}***	***0.91***	***0.13***	***0.46***
		10 914 312	***rs14428120***	***17.3 ± 5.3***	***1.1×10^{-3}***	***0.07***	***0.84***	***0.43***

		2 392 397	***rs14419462***	***23.4 ± 8.1***	***3.8×10^{-3}***	***0.35***	***0.05***	***0.13***
		1 379 849	rs314301286	-42.3 ± 13.3	1.5×10^{-3}	0.04	0.07	0.04
		3 589 044	rs10732133	-36.6 ± 11.1	9.4×10^{-4}	0.28	0.04	0.06
		4 908 910	rs14422558	-151.2 ± 55.9	6.9×10^{-3}	0.00	0.11	0.00
		11 693 052	rs314301286	37.0 ± 14.8	1.3×10^{-2}	0.07	0.18	0.03
		11 345 880	rs14428501	-22.5 ± 9.2	1.4×10^{-2}	0.02	0.34	0.09
		2 850 506	rs14420072	49.5 ± 22.0	2.5×10^{-2}	0.00	0.09	0.01
4	Growth7	***88 325 118***	***rs15639000***	***-21.0 ± 5.4***	***9.9×10^{-5}***	***0.46***	***0.52***	***0.47***
		86 755 267	***rs14499758***	***-28.0 ± 6.3***	***7.9×10^{-6}***	***0.11***	***0.43***	***0.26***
		87 755 983	rs318052788	21.0 ± 8.9	1.8×10^{-2}	0.04	0.18	0.09
		87 290 961	rs14500395	-286.6 ± 138.1	3.8×10^{-2}	0.00	0.02	0.00
5	Growth8	***36 291 277***	***rs13585490***	***-32.9 ± 5.3***	***4.5×10^{-10}***	***0.39***	***0.54***	***0.40***
		38 774 986	***rs315605733***	***-29.6 ± 6.5***	***5.8×10^{-6}***	***0.04***	***0.27***	***0.19***
		35 299 978	***rs16487933***	***-19.9 ± 6.2***	***1.2×10^{-3}***	***0.65***	***0.25***	***0.24***
		36 426 195	rs15697504	-49.3 ± 16.0	2.0×10^{-3}	0.22	0.46	0.03
		38 867 279	***rs314075508***	***-12.6 ± 4.9***	***1.1×10^{-2}***	***0.09***	***0.46***	***0.48***
		36 575 445	rs16489220	-43.4 ± 17.5	1.3×10^{-2}	0.15	0.48	0.02
		34 772 650	***rs16487762***	***14.0 ± 7.0***	***4.6×10^{-2}***	***0.67***	***0.14***	***0.17***
		33 713 055	***rs14530756***	***11.5 ± 5.3***	***3.2×10^{-2}***	***0.22***	***0.68***	***0.35***
7	Growth9	***23 959 214***	***rs16596357***	***-38.9 ± 5.4***	***7.1×10^{-13}***	***0.33***	***0.50***	***0.34***
		26 021 398	rs14618036	80.8 ± 22.6	3.6×10^{-4}	0.11	0.00	0.01
		18 544 622	***rs14611566***	***18.3 ± 5.3***	***5.8×10^{-4}***	***0.50***	***0.50***	***0.46***
		33 699 148	rs16610251	67.0 ± 23.0	3.5×10^{-3}	0.24	0.04	0.01
		32 262 733	***rs317586448***	***-15.1 ± 5.2***	***3.4×10^{-3}***	***0.74***	***0.41***	***0.44***
		27 518 867	rs14619679	32.7 ± 12.1	6.8×10^{-3}	0.09	0.09	0.05
		29 631 963	***rs10727581***	***17.2 ± 6.2***	***5.4×10^{-3}***	***0.39***	***0.07***	***0.22***
		29 999 307	rs14622731	-30.3 ± 10.6	4.3×10^{-3}	0.26	0.00	0.06
		23 134 876	rs312633887	-30.8 ± 12.0	1.0×10^{-2}	0.41	0.18	0.05
		35 390 789	rs317022486	-25.2 ± 10.0	1.2×10^{-2}	0.15	0.30	0.07
		14 071 039	rs16088281	34.6 ± 15.8	2.9×10^{-2}	0.02	0.09	0.03
		25 726 731	rs317769956	-22.5 ± 9.0	1.2×10^{-2}	0.00	0.34	0.09
		24 549 929	rs14616465	-101.8 ± 50.3	4.3×10^{-2}	0.00	0.11	0.00
20	Growth12	***10 667 729***	***rs16172598***	***26.3 ± 5.4***	***1.1×10^{-6}***	***0.67***	***0.11***	***0.33***
		10 965 403	rs16173231	346.1 ± 139.1	1.3×10^{-2}	0.04	0.00	0.00
		8 814 245	rs314843162	21.9 ± 8.8	1.3×10^{-2}	0.24	0.11	0.09
		13 427 530	***rs16176151***	***-13.9 ± 5.2***	***7.0×10^{-3}***	***0.35***	***0.48***	***0.48***
		7 881 700	rs312541071	-73.1 ± 29.7	1.4×10^{-2}	0.20	0.07	0.01
		11 389 861	rs13634923	19.2 ± 8.3	2.1×10^{-2}	0.24	0.20	0.11
		10 165 171	***rs14278292***	***-12.7 ± 5.8***	***2.9×10^{-2}***	***0.50***	***0.13***	***0.26***
		9 302 754	***rs14277526***	***14.1 ± 6.1***	***2.0×10^{-2}***	***0.39***	***0.05***	***0.23***

[a]Gallus Gallus Autosome; [b]QTL name as in (Jacobsson et al. 2005); [c] Base pair position according to *Chicken* genome assembly (galGal3) of May 2006; [d]SNP name as in NCBI dbSNP; [e]Additive effect ± Standard Error calculated within each QTL as described in model 1; [f] Significance of the estimated additive genetic effect from fitting model 1 where the allele-coding is based on the major/minor allele-frequencies and not the line origin of the alleles as in Table 2; [g]Frequency of the allele corresponding to the estimated effect, calculated from all founder birds which originates from the high-weight selection line; [h]Frequency of the allele corresponding to the estimated effect, calculated from all founder birds which originates from the low-weight selection line; [i]Minor allele frequency calculated from all 1348 birds, AIL generation F_2-F_8, included in the study.
